# Molecular treatment effects of alemtuzumab in skeletal muscles of patients with IBM

**DOI:** 10.1186/s12883-016-0568-5

**Published:** 2016-04-16

**Authors:** Karsten Schmidt, Konstanze Kleinschnitz, Goran Rakocevic, Marinos C. Dalakas, Jens Schmidt

**Affiliations:** Department of Neurology, University Medical Center Göttingen, Robert-Koch-Str. 40, 37075 Göttingen, Germany; Department of Neuroimmunology, Institute for Multiple Sclerosis Research and Hertie Foundation, University Medical Center, Göttingen, Germany; Department of Neurology, Thomas Jefferson University, Philadelphia, PA USA; Neuroimmunology Unit, Department of Pathophysiology, University of Athens Medical School, Athens, Greece

**Keywords:** Muscle inflammation, Inclusion body myositis, Alemtuzumab, T-cell depletion

## Abstract

**Background:**

Mechanisms of inflammation and protein accumulation are crucial in inclusion body myositis (IBM). Recent evidence demonstrated that intravenous immunoglobulin failed to suppress cell-stress mediators in IBM. Here we studied the molecular changes in skeletal muscle biopsies from patients with IBM before and after treatment with alemtuzumab.

**Methods:**

Relevant inflammatory and degeneration-associated markers were assessed by quantitative-PCR and immunohistochemistry in repeated muscle biopsy specimens from patients with IBM, which had been treated in a previously published uncontrolled proof-of-concept trial with alemtuzumab.

**Results:**

There were no significant changes of the mRNA expression levels of the pro-inflammatory chemokines CXCL-9, CCL-4, and the cytokines IFN-γ, TGF-β, TNF-α, and IL-1β. Similarly, the degeneration-associated molecules ubiquitin, APP and αB-crystallin did not substantially change. Although no overall beneficial treatment effect was noted except for a 6-month stabilization, some patients experienced a transient improvement in muscle strength. In such responders, a trend towards reduced expression of inflammatory markers was noted. In contrast, the expression remained unchanged in the others who did not experience any change. The expression levels of IL-1β and MHC-I correlated with the positive clinical effect. By immunohistochemistry, some inflammatory mediators like CD8, CXCL-9, and MHC-I were downmodulated. However, no consistent changes were noted for ubiquitin, nitrotyrosin and β-amyloid.

**Conclusions:**

Alemtuzumab showed a trend towards downregulation of the expression of some inflammatory molecules in skeletal muscle of IBM patients but has no effect on several crucial markers of cell stress and degeneration. The data are helpful to explain the molecular treatment effects of future lymphocyte-targeted immunotherapies in IBM.

## Background

Inclusion body myositis (IBM), the most common myopathy in patients over 50 years of age, leads to a progressive muscle weakness and atrophy [1]. Histopathological examination in IBM muscles reveals two major features: inflammation and degeneration [2]. Cytotoxic lymphocytes surround and invade non-necrotic myofibers and proinflammatory chemo- and cytokines, such as CCL-2, CCL-3, CCL-4, CXCL-9, IL-1β, TNF-α and TGF-β are overexpressed. On the other hand, degeneration is present with vacuoles and accumulation of unwanted and misfolded proteins like β-amyloid, phosphorylated tau, TDP43 and α-synuclein [3]. Both processes are relevant to the pathogenesis in IBM and seem to be related to each other [4].

Despite the clearly demonstrated inflammatory pathomechanisms, immunosuppressive treatments failed to show a clear benefit in IBM, including methotrexate, mycophenolate-mofetil and the TNF-α blocker etanercept [5–9]. In controlled studies with IVIG, a significant effect could be recognized on dysphagia [10], but no improvement in limb strength could be observed [11, 12]. In an uncontrolled proof-of-concept study with alemtuzumab in 13 IBM patients, a reduction of their disease progression for up to six months was observed [13]. Alemtuzumab is a monoclonal antibody directed against CD52 and therefore leads to a profound depletion of peripheral lymphocytes [14].

In the present study, we have analyzed the molecular changes in skeletal muscle biopsies from the IBM patients, who participated in the previous trial with alemtuzumab.

## Methods

### Patients and muscle biopsies

We used muscle biopsy specimen from skeletal muscle of the 13 IBM patients, who received alemtuzumab in a previous trial [13]. All patients were treated with a single infusion of alemtuzumab at a dose of 1.2 mg/kg over 4 days not exceeding a total dose of 120 mg. Muscle biopsies were performed prior and 6 months after treatment with alemtuzumab at the same site. In most cases, the biceps was used as standard muscle. Occasionally the quadriceps was chosen, e.g. when the biceps was severely atrophic. IRB-approved consent for use of their samples had been obtained.

### Extraction of RNA and quantitative PCR

For extraction of total RNA from muscle biopsies, the RNeasy kit (Qiagen, Valencia, CA, USA) was used, following the supplier’s instructions. The tissue was homogenized in 350 μl lysis buffer, RNA was subsequently eluted in 30 μl water and stored at -80 °C.

For cDNA synthesis, SuperScript II reverse transcriptase (Invitrogen, Darmstadt, Germany) was used, according to the supplier’s instructions. Originated cDNA was amplified with master mix for real-time PCR (Invitrogen) using 6-carboxy-fluorescein (FAM)-labelled probes and specific primers (Applied Biosystems, Carlsbad, CA, USA): Glyceraldehyde-3-phosphate dehydrogenase (GAPDH, s99999905_m1); APP (Hs00169098_m1), TGF-β1 (Hs00171257_m); IL-1β (Hs00174\097_m1); ubiquitin (Hs00430290_m1); CXCL-9 (Hs00171065_m1); αB-crystallin (Hs00157107_m1); NCAM (Hs00169851_m1); CCL-4 (Hs00605740_g1); IFN-γ (Hs00174143_m1); TNF-α (Hs00174128_m1). Reactions were performed in duplicates on a SDS 7500 Sequence Detection System (Applied Biosystems), following the standard cycle protocol and instructions given by the supplier. Target mRNA-expression was quantified using the c(t) method in relation to expression of glyceraldehyde-3-phosphate dehydrogenase (GAPDH) mRNA as housekeeping gene.

### Staining of muscle tissue

For immunohistochemistry, 5 μm frozen sections of all muscle biopsies were fixed in acetone at -20 °C for 10 min. Primary antibodies were diluted in 1 % BSA. Incubation was performed at room temperature for one hour. Following primary antibodies were used: β-amyloid (mouse clone 6E10, Signet, Dedham, MA); MHC class I (rat clone YTH 862.2 from Serotec, Oxford, UK); APP (rabbit polyclonal from Serotec, Oxford, UK); iNOS (rabbit polyclonal from Chemicon/Millipore, Billerica, MA); NCAM (mouse clone Eric-1 from Labvision/Neomarkers, Fremont, CA); αB-crystallin (rabbit polyclonal from Serotec); IL-1β (rabbit polyclonal from Abcam, Cambridge, USA); CXCL-9 (goat polyclonal from R&D); P-neurofilament (mouse clone SMI-31 from Covance, Princeton, NJ, USA).

Consecutive sections of all patients were double-labelled for 1) CXCL-9 and MHC-I; 2) NCAM and αB-crystallin; 3) IL-1β and β-amyloid; 4) iNOS and SMI-31. Goat-derived Alexa 594 or Alexa 488 were used as secondary antibodies and diluted in 1 % BSA. After mounting in Fluoromount G (Southern Biotech, Alabama, USA), digital photography was performed on a Zeiss Axiophot microscope (Zeiss, Göttingen, Germany). Pictures were taken by a cooled CCD digital camera (Retiga 1300, Qimaging, Burnaby, BC, Canada) and visualized with ImageProPlus software (MediaCybernetics, Bethesda, MD). For quantification, two independant raters were scoring the signal intensity on a scale ranging from 0 to 3 and the mean was defined as semiquantitative IHC-Score.

### Statistics

For statistical analysis (*t*-test, Pearson correlation), *p* < 0.05 was used as significant value and all significant outliers (Grubb’s test) were excluded prior to analysis (Graph Pad Prism V5, San Diego, CA, USA).

## Results

### Partial downmodulation of mRNA levels of inflammatory mediators after alemtuzumab

**Fig. 1:**
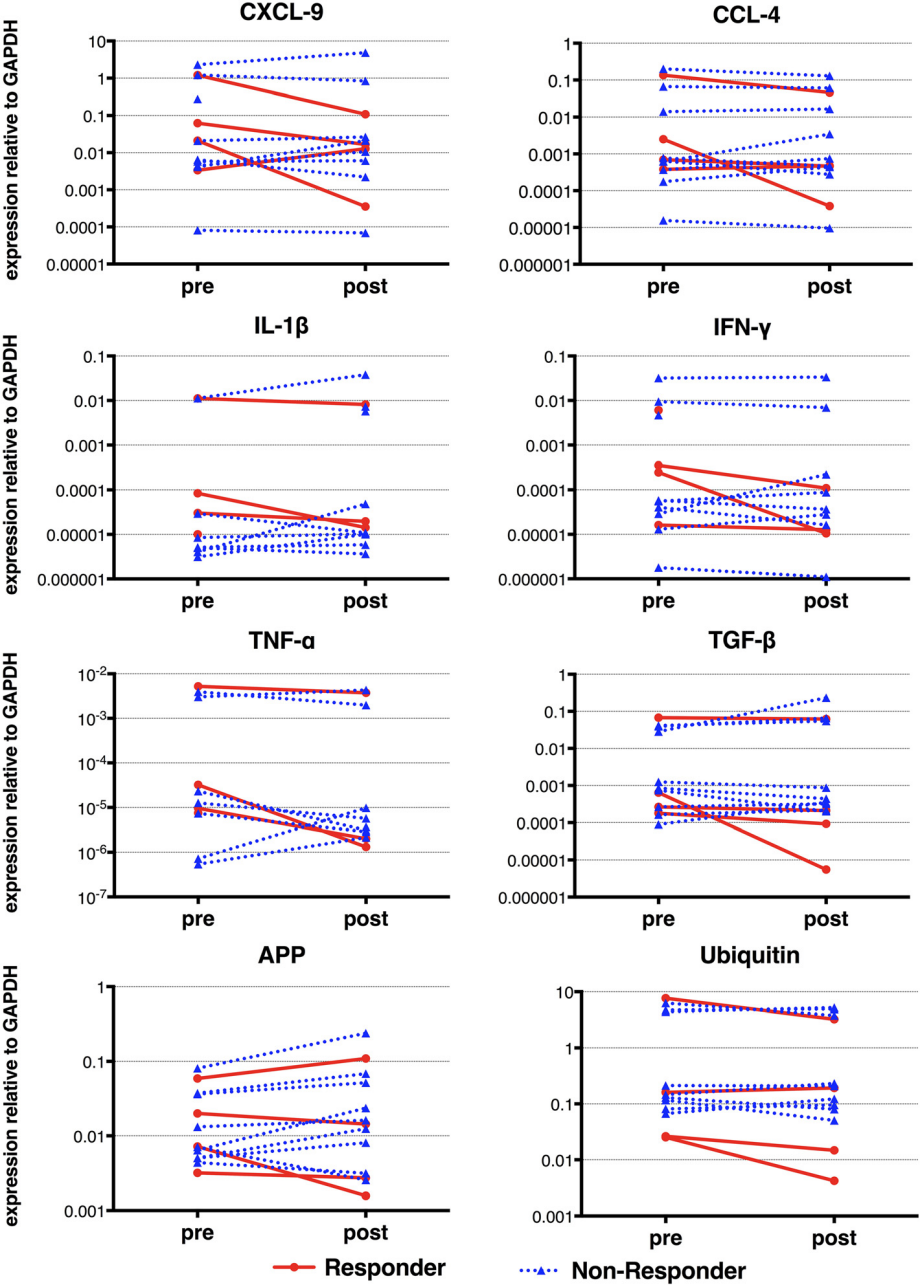
GAPDH-normalized mRNA expression of inflammatory and degenerative markers. The mRNA-expression was assessed by quantitative (real-time) polymerase chain reaction relative to GAPDH in skeletal muscle from patients with inclusion body myositis (IBM) before (pre) and after (post) treatment with alemtuzumab. Subgroups are differentially labeled as responders (red) and non-responders (blue)

The expression levels of several disease-relevant markers of IBM were determined by quantitative PCR (Fig. 1). The mRNA expression of pro-inflammatory chemokines CXCL-9, CCL-4, IL-1β, IFN-γ, and TNF-α were downmodulated after treatment in several patients, but no statistical significance was observed. The expression level of degeneration-associated molecules ubiquitin and APP remained also unchanged before and after treatment.

### Partial reduction of inflammatory markers based on immunohistochemical staining after alemtuzumab

**Fig. 2:**
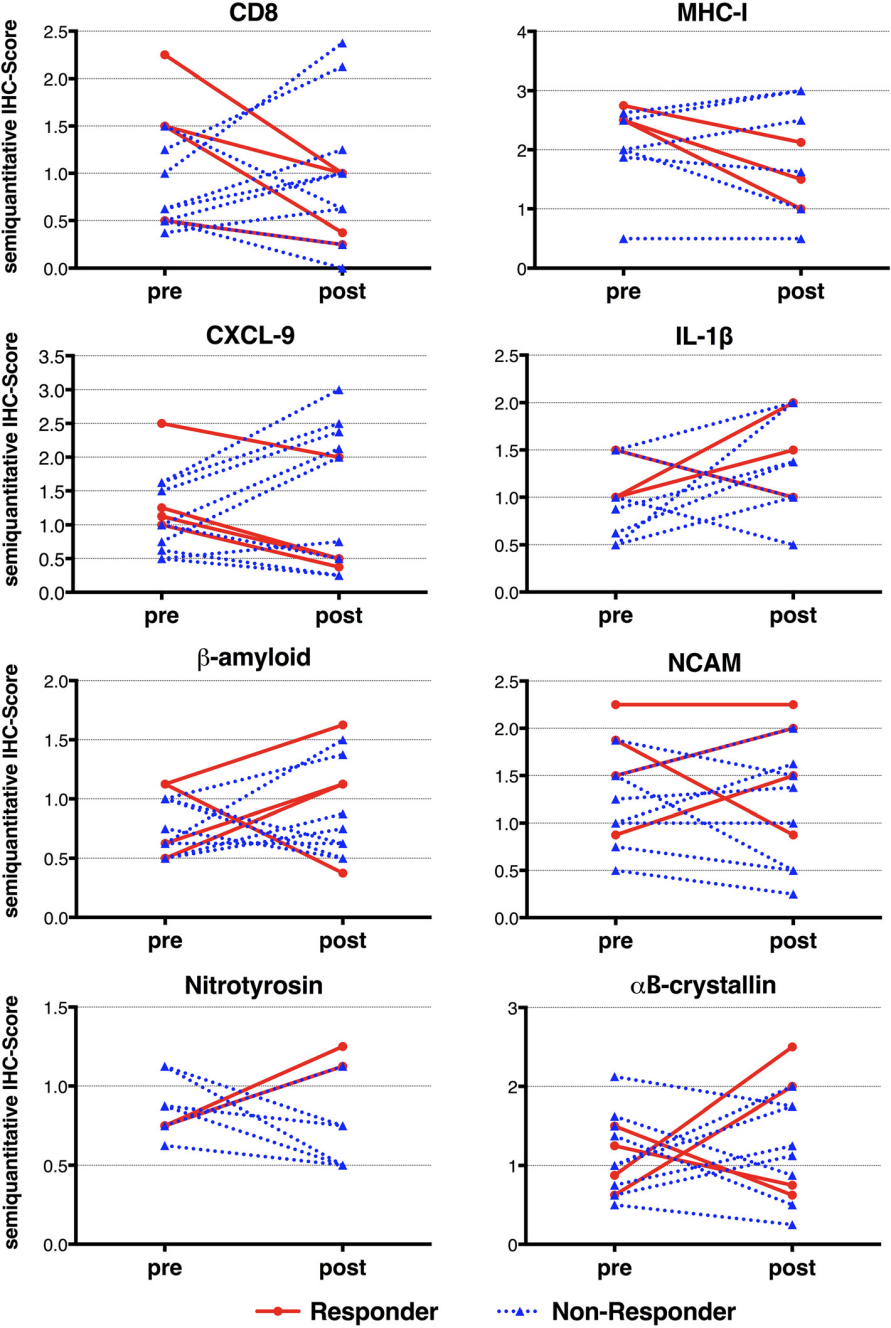
Immunohistochemistry of inflammatory and degenerative markers in skeletal muscle of IBM patients before and after treatment with alemtuzumab. No significant changes were observed for CD8, MHC-I, the proinflammatory chemokine CXCL-9 and the cytokine IL-1β. Similarly, the degeneration-associated molecules nitrotyrosine, β-amyloid, αB-crystallin and NCAM remained without major variation. Subgroups are differentially labeled as responders (red) and non-responders (blue)

In line with the mRNA-expression results, immunohistochemical analysis of relevant pro-inflammatory mediators showed reduced expression levels in several patients, but no statistical significance was reached (Fig. 2). More specifically, CXCL-9 and MHC-I were diminished in the majority of the patients; in contrast, markers for degeneration and cell stress, including β-amyloid and αB-crystallin, remained unchanged after treatment.

### Subtype analysis of patients who experienced a temporary improvement of strength after treatment vs. those who did not

**Fig. 3:**
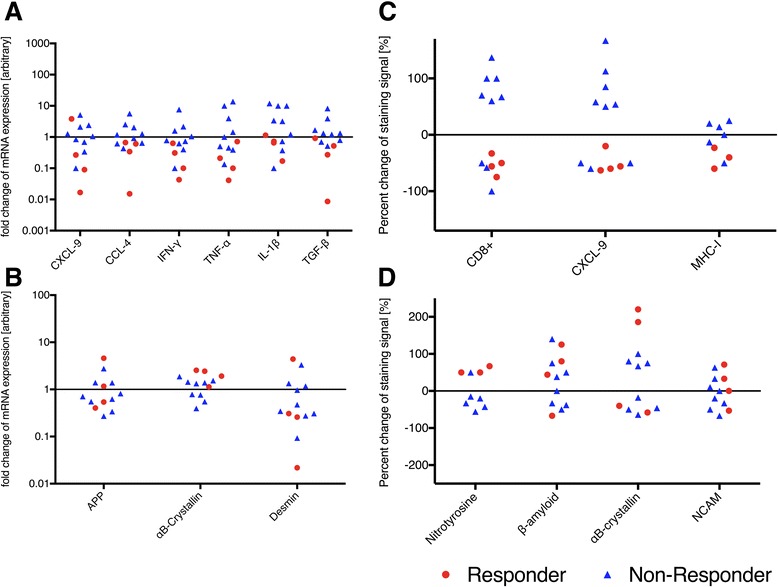
Change of expression of relevant markers measured with qPCR and immunohistochemistry. Mean fold reduction of qPCR assessment (**a**, **b**) and mean reduction in signal intensity (**c**, **d**) before versus after treatment for pro-inflammatory (**a**, **c**) and degeneration-associated molecules (**b**, **d**). Assessment of semiquantitative immunohistochemistry analysis was done by two masked observers of skeletal muscle biopsies stainings from all patients and the mean value is depicted. The group was divided into responders (red) and non-responders (blue). For pro-inflammatory molecules, one can assume a reduction in expression for responders (qPCR: *p* = 0.048 for CCL-4, *p* = 0.0403 for IL-1β; immunohistochemistry: *p* = 0.013 for CD8^+^, *p* = 0.0095 for CXCL-9, *p* = 0.042 for MHC-I), whereas non-responders show an increase of expression of proinflammatory molecules. Only one patient, who responded well to therapy, revealed an increase in CXCL-9 expression. In both groups, the change of expression of degeneration-associated molecules did not differ, regardless of the clinical improvement

Although the study was small and uncontrolled, some patients experienced a clear but short-lasting improvement of weakness in arms and legs [13]. This response was, however, heterogeneous. Four patients experienced a gain of strength at 6 months after treatment as reflected by quantitative muscle testing, MRC scale and subjective improvement; for the purpose of this study, we considered those as “responders”, even if the response is not defined on a statistical basis but only on careful observations collected by the non-treating examiners; in contrast, 9 patients had no clear subjective or objective benefit, and we considered them as “non-responders” [13]. The molecular differences between these two groups were examined and compared with each other. The mRNA expression levels of CXCL-9, CCL-4 (*p* = 0.048), IFN-γ, TGF-β, TNF-α, and IL-1β (*p* = 0.0403) were downmodulated in the “responders” compared to “non-responders” (Fig. 3). By contrast, the expression rate of the de- and regeneration/ cell-stress markers APP, αB-crystallin and desmin displayed a similar expression in both groups (Fig. 3). By immunohistochemical staining, CD8 (*p* = 0.013), CXCL-9 (*p* = 0.0095) and MHC-I (*p* = 0.042) were clearly diminished in the “responders” compared to “non-responders” (Fig. 3); this was in contrast to the protein expression of β-amyloid, NCAM, nitrotyrosine and αB-crystallin which remained unchanged in both groups after treatment (Fig. 3). Accumulation of β-amyloid showed also no difference, regardless of the clinical outcome.

## Discussion

In this study we demonstrate that one series of infusions with alemtuzumab can reduce the expression of pro-inflammatory molecules in some IBM patients, especially those with the most noticeable clinical benefit, suggesting that inflammatory mechanisms in IBM are of clinical importance. Although various immunosuppressants or immunomodulators may transiently help some IBM patients, several short-term controlled trials have failed to demonstrate a beneficial effect, leading to the notion that in IBM, the long-term degenerative process exerts the most deleterious effect compared to autoimmune and inflammatory changes [3]. Our results from the alemtuzumab trial are illuminating because they show suppression of key inflammatory mediators –some of which are connected to enhancing degeneration– in connection with some clinical benefit [13]. Although this was an uncontrolled proof-of-concept study with a small number of patients dictating careful interpretation of data, the noted correlation between the reduction of proinflammatory markers with short clinical benefit in some of the patients, suggests that a potent immunosuppressive treatment, if sustained over longer time periods, may not only alter the proinflammatory muscle microenvironment but may also affect the degenerative process and result in a clinical benefit. One reason for not observing a sustained effect might have been the observation that alemtuzumab did not suppress all the degenerative and cell-stressor molecules. Nitric oxide in particular, a mediator between inflammation and degeneration, was also unchanged.

Similar data we obtained in the repeated muscle specimens in a short-term controlled study with intravenous immunoglobulin and prednisolone, where we demonstrated that inflammatory markers were downmodulated after therapy but not the cell-stress molecules like αB-crystallin and degenerative molecules like APP [15]. Further, compared to the experience with alemtuzumab in MS patients [16, 17], the lack of effect in IBM might be also related to using only one set of infusions since the protocol was designed as a proof-of-concept study rather than a clinical trial. One set of infusions could lead to a different treatment response, considering the repletion kinetics of different lymphocyte subsets [18]. Recently it was shown that regulatory T-cells are reduced in IBM, suggesting a role in pathogenesis [19] especially since they can inhibit the lytic activity of cytotoxic T cells *in vitro* [20]. Because alemtuzumab is reprogramming the immune system [21] and could change the proposed imbalance between cytotoxic and regulatory T-cells, a repeated set of infusions–even more than what is necessary in multiple sclerosis (MS)– may be needed to achieve a sustained effect on specific repletion patterns in order to affect the noxious degenerative molecules.

Another possible explanation for the ineffectiveness or unsustained efficacy of alemtuzumab could be the lack of effect on cytokine expression, especially in those patients who did not experience any clinical benefit. In this case, the upregulation of proinflammatory cytokines may be independent of peripheral T-lymphocytes. Recently, the blocking effect of IL-1β was tested in IBM-patients in a small pilot study of 4 patients, demonstrating no efficacy [22]. Whether there are different subsets of patients with IBM, some of whom may respond differently to an immunosuppressive treatment, cannot be answered so far. In this study, we were not able to identify potential molecular markers that could predict the response to alemtuzumab. Finally, it is possible that the muscle tissue may not be sensitive enough to capture changes of the molecules studied in such a short period.

## Conclusion

In conclusion, our data show that a lymphocyte-targeted immunotherapy can change the pro-inflammatory milieu in some patients with IBM and this may correspond to a better clinical outcome. Most importantly, several crucial markers of cell stress and degeneration remain unchanged, providing an explanation for lack of sustained clinical benefit. In the future, clinical trials examining the molecular inflammatory and degenerative changes in the muscle and correlating them with clinical outcomes may shed light in understanding the pathogenesis of IBM.
